# Intracavitary cardiac metastasis of cervical squamous cell carcinoma with immune thrombocytopenia: a rare case report

**DOI:** 10.3389/fonc.2023.1239606

**Published:** 2023-08-30

**Authors:** Ning Liu, Deguan Lv, Raydonna Rachel Schneider, Hongyan Yang, Mingyan Zhang, Yanan Liu, Meili Sun

**Affiliations:** ^1^ Department of Oncology, Central Hospital Affiliated to Shandong First Medical University, Jinan, China; ^2^ Department of Oncology, Jinan Central Hospital, Shandong University, Jinan, China; ^3^ Division of Hematology/Oncology, Department of Medicine, UPMC Hillman Cancer Center, University of Pittsburgh, Pittsburgh, PA, United States

**Keywords:** cervical cancer, intracavitary cardiac metastasis, immune thrombocytopenia, immune checkpoint inhibitors, circulatory failure

## Abstract

Cervical cancer is a prevalent gynecological malignancy; however, intracavitary cardiac metastasis of cervical squamous cell carcinoma is exceptionally rare. In addition, the co-occurrence of cervical cancer and right ventricular cancer thrombus with autoimmune diseases is extremely uncommon. Furthermore, the role of immune checkpoint inhibitors in the treatment process of such cases remains controversial. Given the scarcity of reported cases, it is imperative to document and highlight this unique presentation, providing novel insights into diagnosis and management strategies. We present the case of an adult patient diagnosed with cervical cancer and concurrent right ventricular cancer thrombus, accompanied by immune thrombocytopenia (ITP). The patient exhibited resistance to conventional ITP drugs, with suboptimal platelet response. However, upon achieving initial control of the tumor, the patient’s platelet counts returned to normal. Notably, the addition of immune checkpoint inhibitors targeting PD-L1 resulted in effective tumor control, accompanied by sustained high platelet levels. Unfortunately, during subsequent anti-tumor therapy, the patient experienced a prolonged platelet rise time, rendering continuous effective anti-tumor therapy and anticoagulant therapy unattainable. This led to a gradual increase in intraventricular thrombosis, ultimately resulting in the patient’s demise due to circulatory failure. This rare case sheds light on the potential alleviation of ITP in patients with tumor complications through effective antitumor therapy. The successful control of ITP after tumor management highlights the importance of integrated treatment approaches. Furthermore, the inclusion of immune checkpoint inhibitors demonstrated their potential role in achieving tumor control and maintaining platelet levels. However, the prolonged platelet rise time observed during subsequent therapy underscores the challenges in maintaining both effective anti-tumor therapy and anticoagulant therapy, necessitating careful management strategies. This case report emphasizes the need for a comprehensive evaluation and tailored therapeutic interventions in similar complex scenarios. In summary, this case report offers valuable clinical insights into the management of intracavitary cardiac metastasis of cervical squamous cell carcinoma, the coexistence of immune thrombocytopenia, and the potential implications of immune checkpoint inhibitors in such cases. Understanding these rare occurrences and their clinical impact can contribute to improved diagnostic approaches, therapeutic decision-making, and patient outcomes.

## Background

Cervical cancer is a common malignancy among women, with squamous cell carcinoma being the most prevalent subtype. Metastasis of cervical cancer can occur through various routes, including direct spread, lymphatic dissemination, and hematogenous dissemination. While metastasis to distant organs is well-documented (1), cardiac metastasis from cervical cancer is relatively rare and has been poorly reported in the literature.

Cardiac tumors, both primary and metastatic, are uncommon, accounting for a small percentage of surgical thoracotomy cases (2). Metastatic tumors are more frequently encountered than primary cardiac tumors, with lung cancer, lymphoma, and breast cancer being the most common primary sources (3). Pericardial metastasis is the most frequent type, followed by epicardial and myocardial involvement. Endocardial metastases are rare, and usually associated with intravascular growing tumors, such as those originating from the kidney, liver, or uterus (4). The diagnosis of cardiac tumors relies on various imaging techniques, including echocardiography, computed tomography (CT), magnetic resonance imaging (MRI), and positron emission tomography-computed tomography (PET-CT). Histopathological examination remains the gold standard for definitive diagnosis, although many cases are confirmed postmortem (5). Treatment options for cardiac malignancies, including metastatic tumors, are limited, and the prognosis is generally poor, with reported survival ranging from several months to a maximum of two years (6).

Immune thrombocytopenia (ITP) is an autoimmune hematologic disorder characterized by decreased platelet counts due to immune-mediated platelet destruction and/or impaired thrombogenesis (7). In some cases, ITP may coexist with malignancies, potentially complicating the management and treatment outcomes. Autoantibodies against platelet-specific antigens, such as glycoprotein (GP) Ib-IX, play a significant role in the pathogenesis of ITP. Patients with ITP and anti-GPIb autoantibodies often experience more severe reductions in platelet counts and exhibit reduced responses to conventional therapies (8, 9).

Considering the rarity of cardiac metastasis from cervical cancer and the unique aspect of concomitant immune thrombocytopenia, this case report aims to provide further insight into the diagnosis and management challenges associated with such cases. By presenting this clinical scenario and reviewing the existing literature, we aim to contribute to understanding this rare entity and emphasize the importance of interdisciplinary collaboration for optimal patient care.

## Case presentation

### Symptoms and diagnosis

In November 2021, an adult patient presented to the Department of Gynecology in our hospital with a chief complaint of irregular vaginal bleeding along with chest tightness after activity for two weeks. The patient had no significant past or family medical history. Upon physical examination, enlarged left neck and right inguinal lymph nodes were observed. The patient’s human papillomavirus (HPV)16 test results were positive for the high-risk type. HPV is recognized as the primary cause of cervical cancer. Tumor markers were negative, except for the enlarged left neck and right inguinal lymph nodes. However, the patient’s blood routine revealed a platelet count of 56*10^9^/L.

On November 8, 2021, the patient underwent bone marrow aspiration and biopsy. The bone marrow cytology results showed low myelodysplastic activity, a normal granulocyte/erythrocyte ratio, and poor megakaryocyte maturation (see **Supplementary Material**, **Figure S1**). Furthermore, the bone marrow biopsy pathology results (see **Supplementary Material**, **Figure S2**) indicated approximately normal bone marrow hyperplasia, a generally normal proportion of granulocytes and red cells, with dominance by young and middle-aged cells. Megakaryocytes with lobulated nuclei were easily observed, exhibiting moderate cell bodies and occasionally less lobulated nuclei. Peripheral blood tests related to immune thrombocytopenia (ITP) demonstrated the following results: reticulated platelet: 11.2% (12.5 ± 4.25%); PAIgG: 25.8% (35.6 ± 7.4%); MAIPA (flow cytometry): GPIb/IX negative, GPIIIa negative, GPIb positive, GPIIb negative, GMPI40 negative, confirming the diagnosis of immune thrombocytopenia. However, there were no significant changes in platelet count following treatment with glucocorticoids and thrombopoietin (TPO), with the highest platelet count reaching 58*10^9^/L.

On November 17, 2021, the patient underwent a biopsy of the left cervical lymph node, revealing metastatic poorly differentiated carcinoma (see **Supplementary Material**, **Figure S3**). Immunohistochemistry results indicated CK5/6 (+), P40 (+), Ki67 (+ about 40%), consistent with poorly differentiated squamous cell carcinoma.

**Figure 1:**
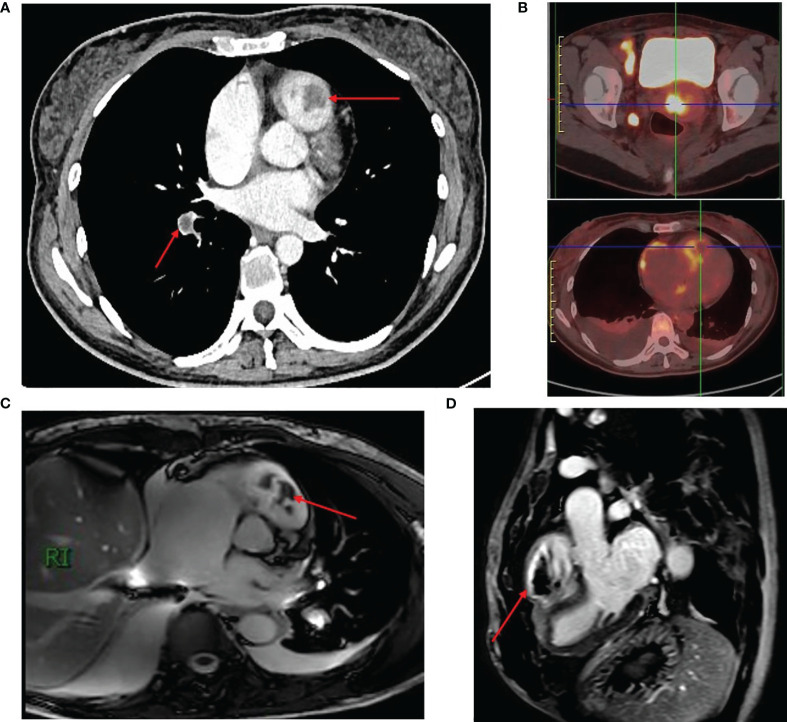
Radiological Findings in a Case of Advanced Cervical Cancer with Right Ventricular Thrombosis and Metastasis. **(A)** Enhanced CT scan showing right ventricular thrombosis and pulmonary embolism. **(B)** PET-CT scan revealed a cervical mass, lymph node enlargement, and tumor thrombus in the right ventricle. **(C, D)** Cardiac MRI demonstrating thrombosis with right ventricular. The red arrows represent right ventricular and pulmonary thrombus.

Further investigations included an enhanced computed tomography (CT) examination of the chest, abdomen, and pelvis on November 18, 2021 (**Figure 1A**). The CT scan revealed right ventricular multiple thrombosis, right lower pulmonary embolism, right atrium enlargement, and widening of the inferior vena cava. Multiple enlarged lymph nodes were observed in the retroperitoneum and pelvic cavity. Additionally, increased uterine volume and cystic changes in bilateral adnexal areas were noted.

To further assess the right ventricular lesions, a cardiac color Doppler ultrasound was conducted on November 19, 2021. The ultrasound revealed large right atrium, continuous hyperechogenicity on the endocardial surface of the right ventricle, involvement of the tricuspid valve leaflets and right ventricular outflow tract, combined with tricuspid regurgitation and mild pulmonary hypertension.

Subsequent PET-CT imaging on November 23, 2021 (**Figure 1B**) considered that the tumor derived from cervical squamous epithelial cells. The scan revealed a cervical mass with enlarged lymph nodes in the right inguinal region, right pelvic wall, bilateral para vascular iliac, retroperitoneal, and left neck. Moreover, tumor thrombus formation in the right ventricle and right lower pulmonary artery, and bilateral pleural metastasis with hydrothorax, were suspected.

November 29, 2021, we provided further insights into the patient’s condition (**Figures 1C, D**). The cardiac MR examination showed a significantly enlarged right atrium, enlarged right ventricle with right ventricular dysfunction, right ventricular apical occlusion, uneven thickening of the right ventricular wall, widening of the right ventricular outflow tract, and fibrosis of the right ventricular myocardium. The examination also revealed an enlarged tricuspid annulus with thickened leaflets and a mural thrombus. Thrombosis was observed in the right ventricular chamber and right ventricular outflow tract, with a wide base attached to the right ventricular apex. These findings were consistent with restrictive right ventricular cardiomyopathy, obstructive cardiomyopathy (right ventricular type), and thrombosis with right ventricular dysfunction. Furthermore, a contrast echo examination was conducted, revealing significant microbubble filling with contrast agent observed in the right intracardial, tricuspid, and pulmonary root echoic masses. These findings strongly suggest the likelihood of tumor metastasis.

Overall, the case involves an adult patient with cervical cancer who presented with irregular vaginal bleeding and chest tightness. The patient’s medical history was unremarkable, and the physical examination revealed enlarged lymph nodes. Further investigations confirmed the presence of poorly differentiated squamous cell carcinoma in the lymph nodes. The patient also exhibited immune thrombocytopenia, with persistently low platelet counts despite treatment. Imaging studies revealed the involvement of the right ventricle, including thrombosis and cardiac dysfunction.

This case presents a unique and rare combination of cervical cancer, immune thrombocytopenia, and intracavitary cardiac metastasis. The diagnostic challenges and management complexities associated with these conditions make this case noteworthy. It highlights the importance of a multidisciplinary approach and the potential implications for treatment strategies in similar cases.

### Treatment process

When the tumor thrombus in the right ventricle and right inferior artery was detected by enhanced CT, we initiated multidisciplinary treatment (MDT) immediately. According to multidisciplinary advice, rivaroxaban was selected for anticoagulation therapy (started with a load dose of 15mg bid d1-21, followed by 20mg qd maintenance). However, during the process of definitive diagnosis, the patient experienced rapid disease progression, worsening chest distress, and suffocation. Although no pleural effusion was observed during the initial enhanced CT examination on November 18, 2021, bilateral pleural effusion appeared in the PET-CT examination five days later (**Figure 1B**). Given the patient’s advanced cervical cancer with immune thrombocytopenia, surgical options for treating the right ventricular tumor thrombus, such as cardiac and vascular surgery, was ruled out. As a result, the patient was referred to the oncology department for further treatment.

Considering the strong desire of the patient and their family for anti-tumor therapy, the patient received a combination of 75 mg/m^2^ Docetaxel on Day 1 and 25 mg/m^2^ Cisplatin on Days 1 to 3, starting on November 26, 2021. On the sixth day after chemotherapy, the patient’s platelet count dropped to 43*10^9^/L, reaching a nadir of 21*10^9^/L on the eleventh day. With the administration of TPO therapy, the platelet count increased to 73*10^9^/L on the nineteenth day of chemotherapy (December 14, 2021). Despite one cycle of chemotherapy, the patient continued to experience chest distress and suffocation. A thoracentesis drainage procedure was performed on the right side on December 14, 2021, to alleviate the symptoms of bilateral massive pleural effusion. Pleural fluid cytology did not detect any cancer cells.

**Figure 2:**
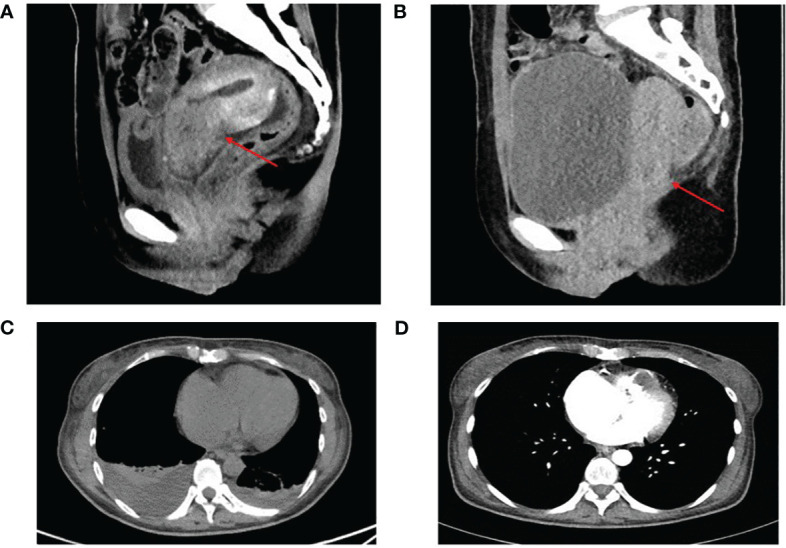
The cervical lesion and bilateral pleural effusion before **(A, C)** and after **(B, D)** 2 cycles of treatment. Partial response of the lesion in the cervical (**A** vs **B**). The bilateral pleural effusion was reduced (**C** vs **D**). The red arrow shows the cervical lesion.

In 2021, the European Society for Medical Oncology (ESMO) reported the results of KEYNOTE-826, which demonstrated that the addition of pembrolizumab treatment significantly prolonged median progression-free survival (mPFS) in patients with advanced cervical cancer and programmed death-ligand 1 (PD-L1) combined positive score (CPS) greater than 1, 10.4 months versus 8.2 months (10). This marked the beginning of immunotherapy for cervical cancer. Subsequent clinical studies have explored PD-1/PD-L1 immune checkpoint inhibitors such as nivolumab, durvalumab, and atezolizumab. In the present case, the patient’s tumor tissue samples were sent for testing to Qiu Zhen Medical Technology Co., LTD., where the expression of pan-oncogene and PD-L1 was detected using the next-generation sequencing (NGS) method. The results, received on December 07, 2021, indicated a tumor mutational burden (TMB) of 25.27 Mut/Mb, microsatellite stability (MSI): MSS, and PD-L1 immunohistochemistry CPS of 94 (**Supplementary Material**, **Figure S4**). Based on these findings, atezolizumab in combination with the TP regimen was added to the patient’s antitumor therapy on December 19, 2021. The patient experienced a minimum platelet count of 25*10^9^/L on Day 10 after treatment, which increased to 81*10^9^/L with TPO treatment. Following this cycle of treatment, the patient underwent a CT reexamination, revealing a significant reduction in bilateral pleural effusion and a decrease in the size of the cervical lesion (**Figure 2**). The treatment effect was assessed as stable, with a tendency toward reduction.

This treatment regimen was continued for four cycles, with TPO used prophylactically to elevate platelet levels after each treatment. During treatment, the patient’s platelet levels fluctuated, reaching a minimum of 25*10^9^/L and increasing to 126*10^9^/L. As treatment progressed, the patient experienced relief from chest tightness and suffocation. She could take care of herself and rest at home during the interval of treatment, which greatly improved the quality of life of the patient, and the patient was very satisfied with the treatment plan. After completing six cycles of combined chemotherapy, there were no significant changes in the right ventricular tumor thrombus, but the primary cervical tumor and metastatic lymph nodes showed a reduction, and bilateral pleural effusion resolved. The overall tumor response was evaluated as a partial response.

Considering the prolonged recovery time of platelets after six cycles of combined chemotherapy and the declining tolerance of the patient to tumor therapy, a maintenance therapy plan was implemented, which involved continuing with atezolizumab immunotherapy. Starting on April 11, 2022, the patient received palliative radiotherapy to reduce the tumor burden at the primary site. Subsequently, external beam radiotherapy was administered to the pelvis at a dose of 50.4 Gy/1.8 Gy/28 f. On April 15, 2022, the patient’s platelet count was 99*10^9^/L but showed a persistent decline to as low as 24*10^9^/L, leading to the suspension of radiotherapy after eight doses. To address the declining platelet levels, TPO was administered, but with poor effect, resulting in a decrease to 7*10^9^/L. The patient received repeated platelet transfusions, glucocorticoids, and a combination of eltrombopag and TPO to promote platelet production. However, the platelet levels remained low, and additional treatments such as gamma globulin and tretinoin were administered. Unfortunately, the patient developed dyspnea, with elevated B-type natriuretic peptide (BNP) levels reaching 2058.22 pg/ml. We promptly sought a cardiology consultation and based on the recommendations, we initiated a treatment plan comprising diuretics (spironolactone, furosemide) for diuresis, medications to enhance heart function (Entresto), and nutritional myocardial agents (coenzyme Q10).

Cardiac MRI conducted on May 18, 2022, revealed a significant enlargement of the right atrium, with thrombus formation in the right ventricular ventricle and right ventricular outflow tract, particularly in the right ventricular outflow tract. Echocardiography on May 20, 2022, showed abnormal echo signals in the right ventricle (suggesting the presence of a tumor), moderate-to-severe tricuspid regurgitation, and pulmonary hypertension. The patient’s dyspnea gradually worsened, and they succumbed to circulatory failure on June 5th, 2022. The overall survival from the start of the patient’s antineoplastic therapy was 193 days.

Throughout the treatment process, **Supplementary Material**, **Figure S5**, provides a schematic representation of the patient’s platelet changes.

In conclusion, the treatment approach for this case of advanced cervical cancer with immune thrombocytopenia involved a combination of chemotherapy, immunotherapy, and palliative radiotherapy. Despite a partial response in the cervical lesion and resolution of bilateral pleural effusion, the patient experienced declining platelet levels and worsening dyspnea, ultimately leading to circulatory failure. This case highlights the challenges in managing advanced cervical cancer, particularly in patients with immune thrombocytopenia, and underscores the need for further research to improve treatment strategies and outcomes in this population.

## Discussion

Cervical cancer is a prevalent gynecological malignancy, primarily affecting women between the ages of 40 and 70 in China. Metastasis in cervical cancer can occur through direct spread, hematogenous dissemination, or lymphatic pathways. However, reports of cardiac metastasis in cervical cancer cases are scarce (11).

Metastatic tumors are more commonly observed in the heart compared to primary cardiac tumors, with lung cancer, lymphoma, and breast cancer being the most frequent primary sources. Cardiac metastases often involve the pericardium, followed by the epicardium and myocardium. Endocardial metastases are rare, typically located in the right heart, and associated with intravascular growing tumors, such as those originating from the kidney, liver, or uterus.

The prognosis for cardiac malignancies is generally poor, with reported survival ranging from 7 months to a maximum of 2 years from the time of diagnosis (12–14). Diagnosis of cardiac tumors relies on imaging techniques like echocardiography, CT, MRI, and PET-CT. Histopathology remains the gold standard for definitive diagnosis, although it is frequently confirmed post-mortem. However, FDG-PET/CT has shown high sensitivity in distinguishing between benign and malignant cardiac tumors (15).

Treatment of cardiac metastases from cervical cancer is challenging, and there is no standardized approach at present. Palliative measures are often employed due to the advanced stage of the disease and widespread tumor dissemination. Surgical resection is commonly used for primary cardiac tumors and certain benign tumors (16). Mio et al. reported a case involving intracardiac metastasis from an unknown uterine cervical cancer accompanied by severe thrombocytopenia (17). Similarly, both of our cases describe patients who were diagnosed with cardiac metastasis from cervical cancer and presented with severe thrombocytopenia, which is considered a rare occurrence. Both our studies highlight the importance of customized therapeutic interventions and comprehensive evaluations in effectively managing these complex scenarios. In Mio’s report, this patient’s cervical cancer had metastasized to the right atrium, but in our case, it had metastasized to the right ventricle. Ventricular metastasis of malignant tumors is extremely rare. Luckily, the patient in Mio’s report underwent emergency cardiac surgery after the detection of a large mobile mass in the right atrium. Unfortunately, surgical options were not viable due to our patient’s condition. In addition, the patient’s thrombocytopenia in Mio’s report was believed to be associated with the cardiac metastasis. But in our case, the patient’s thrombocytopenia was primarily associated with immune thrombocytopenia (ITP).

In the presented case, due to extensive metastasis and the presence of tumor emboli in the heart, systemic palliative anti-tumor treatment and symptomatic management were recommended after a MDT consultation. Although the use of anticoagulation to prevent embolic events lacks supporting data, considering the patient’s hypercoagulable state and the increased risk of embolism associated with intraventricular tumors, anticoagulant therapy with rivaroxaban was administered in addition to antitumor therapy.

The patient in this case was diagnosed with cervical cancer accompanied by a right ventricular tumor thrombus and concomitant immune thrombocytopenia (ITP). ITP is an autoimmune hematologic disorder characterized by the activation of autoreactive T and B cells, resulting in immune-mediated destruction of platelets and impaired thrombogenesis (7).

ITP primarily involves the dysregulation of autoimmune tolerance, leading to the production of autoantibodies against platelet-specific antigens (18). Approximately 60% of ITP patients exhibit detectable autoantibodies, with integrin αIIbβ3 and glycoprotein (GP) Ib-IX being common targets (18–20). Anti-GPIb/IX antibodies induce platelet clearance through Fc-independent phagocytosis by liver macrophages (21, 22). Patients with anti-GPIb autoantibodies often experience severe reductions in platelet counts and show limited response to traditional treatments such as hormonal therapy and immunoglobulin therapy (8, 9).

In this case, the patient’s bone marrow aspiration revealed low-level hyperplasia and poor maturation of giant cells. Additionally, GPIb was positive in the peripheral blood, indicating both platelet production inhibition and increased platelet destruction. Consequently, the treatment of glucocorticoid and thrombopoietin (TPO) failed to significantly increase platelet counts, with a maximum value of 58*10^9^/L. However, after effectively controlling the cervical tumor with antitumor drugs, the patient’s platelet levels were restored to a maximum of 126*10^9^/L. Subsequent cycles of TP chemotherapy resulted in fluctuations in platelet counts, with the lowest value reaching 21*10^9^/L and the highest increasing to 73*10^9^/L. Despite this, pleural effusion was not adequately controlled, necessitating thoracentesis to alleviate chest distress.

Based on the patient’s NGS test results, the addition of the PD-L1 immune checkpoint inhibitor, atezolizumab, effectively controlled bilateral pleural effusion, and the patient’s platelet counts increased to a maximum of 126*10^9^/L.

From these observations, it can be speculated that the diagnosis of ITP in this patient may be partly attributed to decreased platelet production or increased destruction due to inhibitory cytokines or certain antibodies produced by tumor cells. As effective antitumor therapy reduced tumor cell apoptosis and weakened inhibitory factors, platelet counts significantly increased. However, with subsequent cycles of antitumor therapy, platelet recovery time was prolonged which may be caused by myelosuppression, hindering the continuity of effective anticoagulant therapy. Consequently, intraventricular thrombosis gradually worsened, and brain natriuretic peptide (BNP) levels continued to rise until the patient succumbed to circulatory failure. We obtained written informed consent from the patient’s parents in order to publish the article.

In this particular case, systemic palliative anti-tumor treatment, symptomatic management, and anticoagulant therapy with rivaroxaban were administered for ventricular metastasis. For concomitant immune thrombocytopenia, the addition of atezolizumab, an immune checkpoint inhibitor, proved effective in controlling pleural effusion and increasing platelet counts. Further research and studies are warranted to explore the underlying mechanisms and identify optimal treatment approaches for similar cases involving ventricular metastasis and tumor-associated immune thrombocytopenia.

In summary, this case highlights the rarity of ventricular metastasis in cervical carcinoma and the challenging management it poses. The interdisciplinary approach involving cardiac surgeons and oncologists is crucial in decision-making regarding treatment. Additionally, the case underscores the complex interaction between tumor progression, immune dysregulation, and platelet function in patients with tumor-associated ITP. Understanding these interactions is important for optimizing treatment strategies and improving patient outcomes.

## Data availability statement

The original contributions presented in the study are included in the article/**Supplementary Material**. Further inquiries can be directed to the corresponding author.

## Ethics statement

The studies involving human participants were reviewed and approved by Central Hospital Affiliated to Shandong First Medical University Institutional Clinical Care and Use Committee. The patients/participant's parents provided their written informed consent to participate in this study. Written informed consent was obtained from the individual's parents for the publication of any potentially identifiable images or data included in this article.

## Author contributions

MS, NL, and DL conceptualized the study and drafted and edited the manuscript. RS generated the image data. HY, MZ, and YL analyzed the data and generated pathology reports. All authors approved the final manuscript.
