# Supplementary material for: Intracavitary cardiac metastasis of cervical squamous cell carcinoma with immune thrombocytopenia: a rare case report
Source: Front Oncol. 2023 Aug 30;13:1239606. doi: 10.3389/fonc.2023.1239606 (PMC10499513; doi:10.3389/fonc.2023.1239606)
Supplement: Supplementary file 1 [file DataSheet_1.docx]

Supplementary Material

Intracavitary Cardiac Metastasis of Cervical Squamous Cell Carcinoma with Immune Thrombocytopenia: A Rare Case Report

**Ning Liu^1,3*^, Deguan Lv^2*^, Hongyan Yang^1^, Raydonna Rachel Schneider^3^, Mingyan Zhang^3^, Yanan Liu^3^, Meili Sun^1,3 #^**

**^#^Correspondence:** Meili Sun, smli1980@163.com

## Supplementary Figures


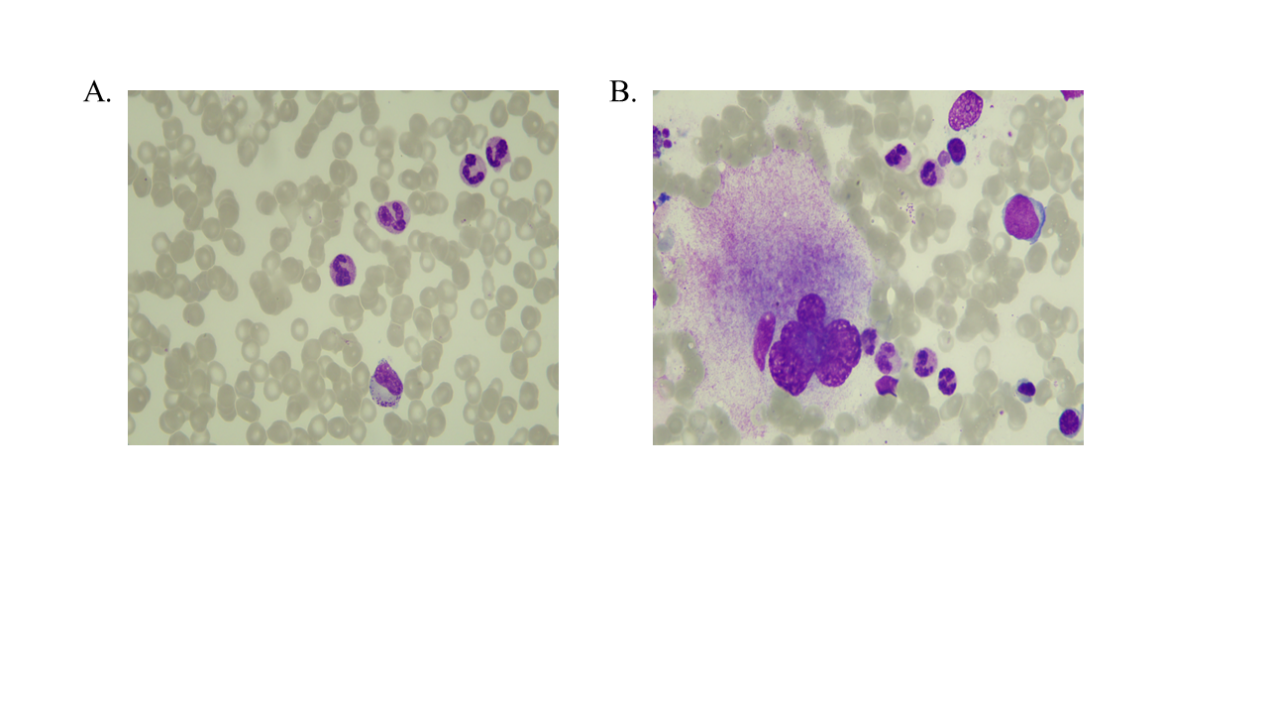


**Figure S1. Bone marrow cytology results.** (A) Low myelodysplastic activity. (B) Poor maturation of megakaryocytes.


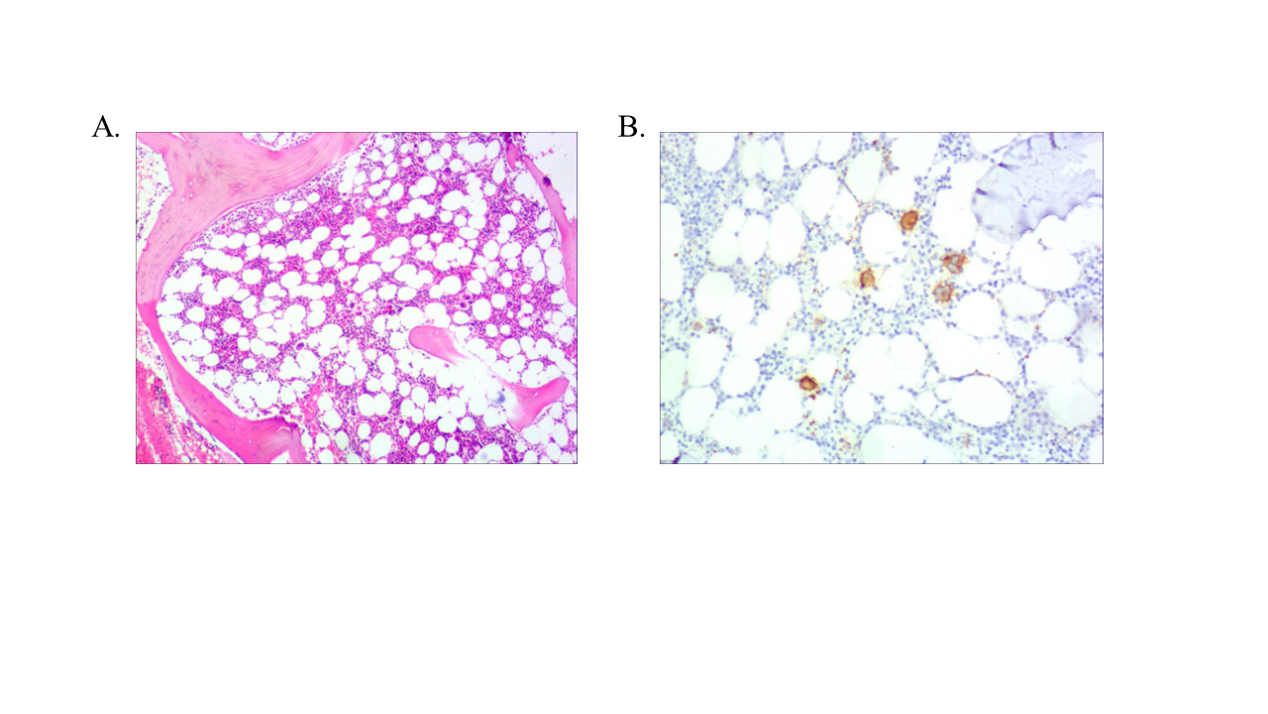


**Figure S2. Bone marrow pathology**. (A) Hematoxylin-eosin staining of bone marrow. (B) Immunohistochemical staining of CD61.


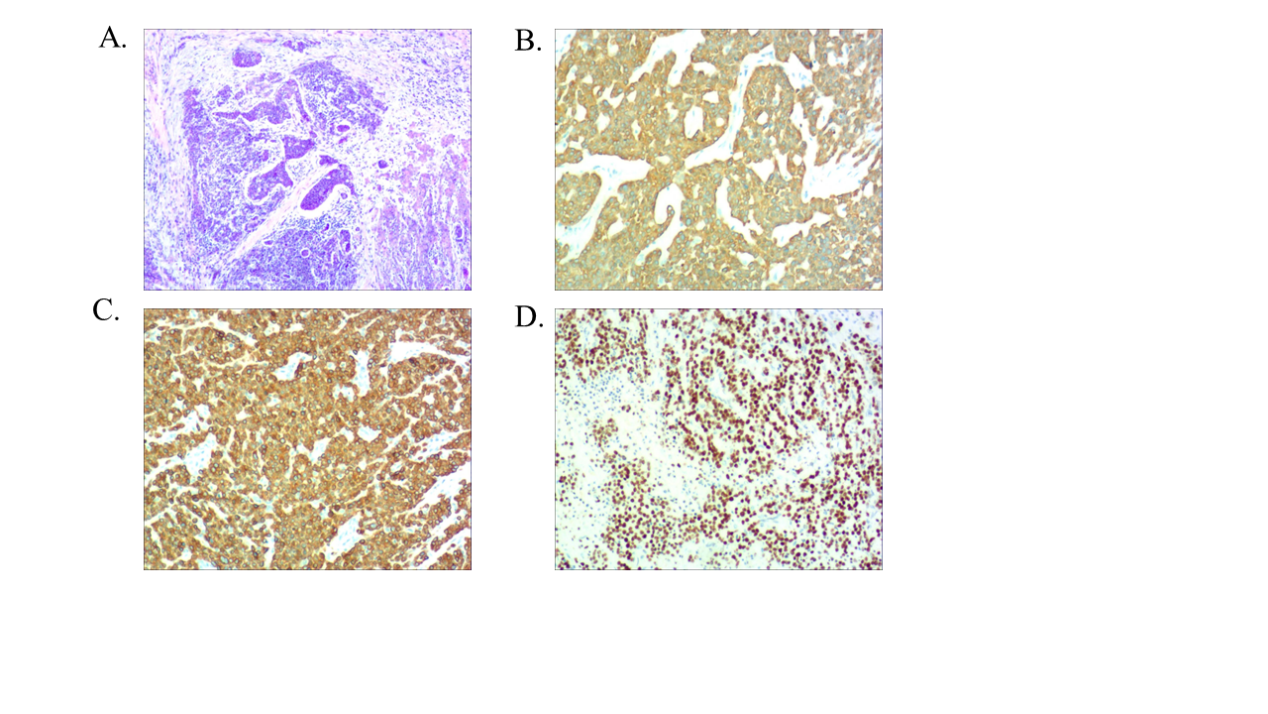


**Figure S3. Lymph node biopsy pathology.** (A) Confirmation of metastatic poorly differentiated carcinoma. (B-D) Immunohistochemistry showed positive staining for CK, CK5/6, and P40, consistent with poorly differentiated squamous cell carcinoma.

**Figure S4.** **Immunohistochemical staining of PD-L1 (200X)**


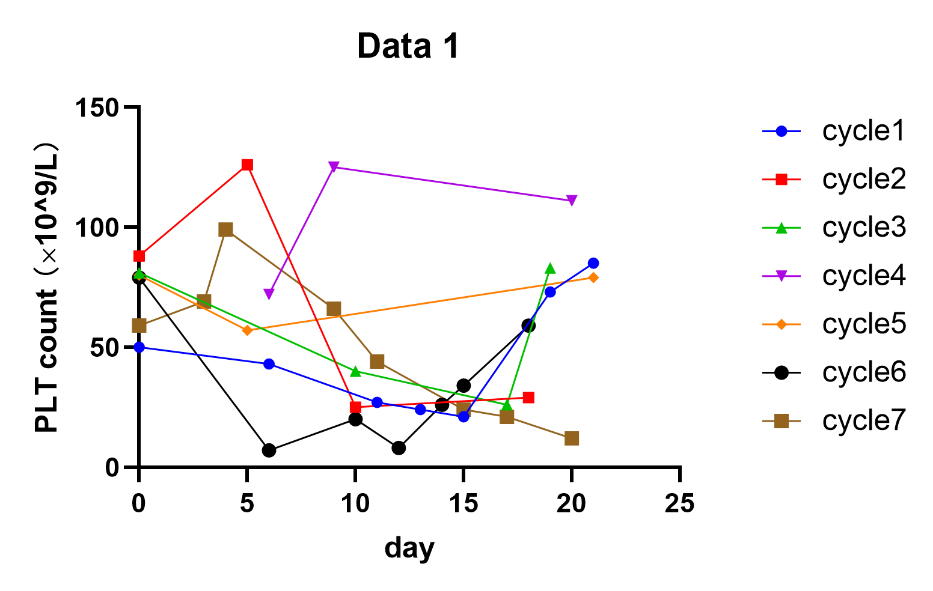


**Figure S5.** **Platelet count changes during each treatment cycle. (PLT, platelet)**.
